# Quality reporting of randomized controlled trials on SGLT2 inhibitors for heart failure: a comprehensive assessment

**DOI:** 10.1038/s41598-024-57514-z

**Published:** 2024-03-21

**Authors:** YueGuang Yang, ShunWen Yang, YuBo Han, GuoLiang Zou, RuiNan Wang, Li Liu

**Affiliations:** 1 Heilongjiang University of Chinese Medicine, Harbin, Heilongjiang 150040 People’s Republic of China; 2 The First Department of Cardiovascular, First Affiliated Hospital, Heilongjiang University of Chinese Medicine, 26 Heping Road, Xiangfang, Harbin, Heilongjiang 150040 People’s Republic of China

**Keywords:** Heart failure, Clinical trial design

## Abstract

Randomised controlled trials (RCTs) provide clinicians with the best evidence of the effectiveness of an intervention, and complete and transparent trial reports help to critically assess and use trial results. The objective of our study was to assess the quality of reporting in RCTs of sodium-glucose co-transporter protein 2 (SGLT2) inhibitors for heart failure (HF) and identify factors associated with improved reporting quality. Two researchers conducted a comprehensive search in four databases (PubMed, Web of Science, EMBASE, and Cochrane). The quality of each report was assessed using a 25-point Overall Quality Score (OQS) based on the guidelines provided in the 2010 Consolidated Standards for Reporting of Trials (CONSORT) statement. We included a total of 58 relevant RCTs. The median OQS in the 2010 CONSORT statement was 15 (range 7.5–24). The missing items were primarily found in the 'Methods' and 'Results' sections of the 2010 CONSORT statement. Multivariate regression modeling revealed that a more recent publication year, high impact factor, and large sample size were significant predictors of OQS improvement. The findings suggest that the overall quality of reported RCTs of SGLT2 inhibitors in HF is unsatisfactory, which reduces their potential usefulness.

## Introduction

Randomised controlled trials (RCTs) are universally acknowledged as the pinnacle of research in evidence-based medicine^1^. RCTs that are meticulously designed and executed can significantly reduce bias, offering direct and robust evidence to inform evidence-based decision-making by clinicians and policymakers^2^. However, RCTs that are suboptimally designed, implemented, or insufficiently reported can compromise the reliability of the trial outcomes, adversely affecting standard clinical practice^3–5^. This scenario can also deteriorate the quality of systematic reviews and meta-analyses^6^. Consequently, it is imperative to ensure the high reporting quality of randomised controlled trials to furnish readers with an exhaustive, lucid, and transparent comprehension of the trial's methodology and implementation process. This enables a critical assessment and utilization of the trial outcomes, ultimately augmenting the precision of clinical decision-making.

The Consolidated Standards for Reporting of Trials (CONSORT) statement, first introduced in 1996^7^ and subsequently revised in 2001^8^ and 2010^9^, offers comprehensive checklists and detailed flowcharts for reporting RCTs. These instruments aid in assessing the utility, reproducibility, and transparency of trials, concurrently mitigating the risk of selective non-disclosure of trial results. The foremost objective is to advocate for transparent and exhaustive reporting of trials, thereby enabling a thorough evaluation and interpretation of the findings.

Sodium-glucose co-transporter protein 2 (SGLT 2) inhibitors were originally engineered to manage type 2 diabetes^10^. Their mechanism involves inhibiting the SGLT2 protein in the kidneys, thereby reducing glucose reabsorption and effectively lowering blood glucose levels. Nevertheless, a number of pivotal trials have demonstrated substantial cardiovascular benefits associated with these inhibitors^11^. These inhibitors can markedly diminish the risk of heart failure (HF) events and cardiovascular mortality^12–14^, including in patients without diabetes^15^. Consequently, SGLT 2 inhibitors have been recognized as a novel therapeutic class for HF management and are endorsed by diverse national treatment protocols in cardiology^16,17^. The aim of this study was to evaluate the reporting quality of published RCTs on SGLT 2 inhibitors for HF in accordance with the CONSORT statement, to pinpoint critical issues and analyse potential underlying causes, thereby offering dependable evidence for future related research and meta-analyses.

## Methods

### Search strategy

An exhaustive systematic literature search was performed across PubMed, Embase, Web of Science, and Cochrane Library databases to pinpoint studies adhering to predefined criteria. The search timeframe extended from the inception of each library to November 2022, exclusively focusing on English-language publications and restricting the study type to RCTs. MeSH headings were utilized for the search process, with PubMed specifically employing search terms such as: [sodium-glucose co-transporter inhibitor OR SGLT2 inhibitor OR SGLT-2 inhibitor OR SGLT 2 inhibitor OR tofogliflozin OR sotagliflozin OR empagliflozin OR canagliflozin OR dapagliflozin OR ertugliflozin OR luseugliflozin OR ipragliflozin OR remogliflozin OR sergliflozin] (term 1); [heart failure OR cardiac failure OR CHF] (term 2). These terms were then strategically combined. The authors of this study declare no involvement in any previous RCTs pertinent to this topic.

### Eligibility criteria

Inclusion Criteria: (1) The study must encompass a population comprising patients diagnosed with HF. (2) The intervention for the test group must involve administration of an SGLT 2 inhibitor. The control group may receive treatments such as a placebo, alternative therapies, varying dosages of the same treatment, or standard care. (3) The study in question must be a RCT. Exclusion Criteria: (1) Trials involving non-human subjects; (2) Studies that are duplicates; (3) Systematic reviews, conference proceedings, meta-analyses, among others; (4) Studies for which the full text is not accessible.

### Quality assessment

Data screening and extraction were performed by two reviewers (YYG and YSW) utilizing a standardized assessment checklist. In instances of differing opinions between the two reviewers, a third reviewer (HYB) intervened to resolve the disagreement. Cohen's kappa statistic was utilized to evaluate the concordance among reviewer ratings (0.66)^18^. This study extends the methodology of a preceding study^19–22^, employing the Overall Quality Score (OQS) to appraise the adherence to the CONSORT statement in each selected paper. The OQS encompasses 25 primary entries as delineated in the 2010 CONSORT statement, 12 of which are subdivided into two sections, culminating in a comprehensive total of 37 secondary entries. A score of 1 was allotted for each reported Level 1 entry, 0.5 for each Level 2 entry, and 0 for entries reported unclearly or not specified. The aggregated scores of all 37 entries were computed to yield a total CONSORT score, ranging from 0 to 25.

### Risk of bias assessment

To evaluate the reliability of the included RCTs as evidence supporting clinical decision-making in HF treatment, the Cochrane Risk of Bias Tool 2.0 (RoB 2.0) was utilized. RoB 2.0 utilizes predefined criteria to appraise studies, categorizing them into 'low risk', 'unclear risk', or 'high risk' for bias. The assessment relies on predetermined criteria that scrutinize the study design and its applicability. The assessment of the risk of bias is conducted by a designated reviewer (WRN).

### Statistical analyses

Descriptive statistical analyses were conducted utilizing SPSS version 24.0. Categorical variables were represented as frequencies (n) and percentages (%), while reported scores were articulated as means and standard deviations (SD). Independent t-tests and one-way ANOVA were employed to assess differences in general characteristics, given that the data adhered to normality and homogeneity. Multivariate linear regression analyses were utilized to investigate characteristics of the tests correlated with report quality. Potential predictors were encoded as follows: region—Europe (Z1 = 0, Z2 = 0, Z3 = 0), North America (Z1 = 1, Z2 = 0, Z3 = 0), Asia (Z1 = 0, Z2 = 1, Z3 = 0), Multi-Regional (Z1 = 0, Z2 = 0, Z3 = 1); fund—no = 0, yes = 1; Journal Impact Factor—< 10 = 0, ≥ 10 = 1; The control group interventions—Empagliflozin (Z1 = 0, Z2 = 0), Dapagliflozin (Z1 = 1, Z2 = 0), Other (Z1 = 0, Z2 = 1); Sample size—0–400 = 0, ≥ 400 = 1; Multicentre trial—No = 0, Yes = 1.

## Results

### Included RCTs and detailed characteristics

**Figure 1:**
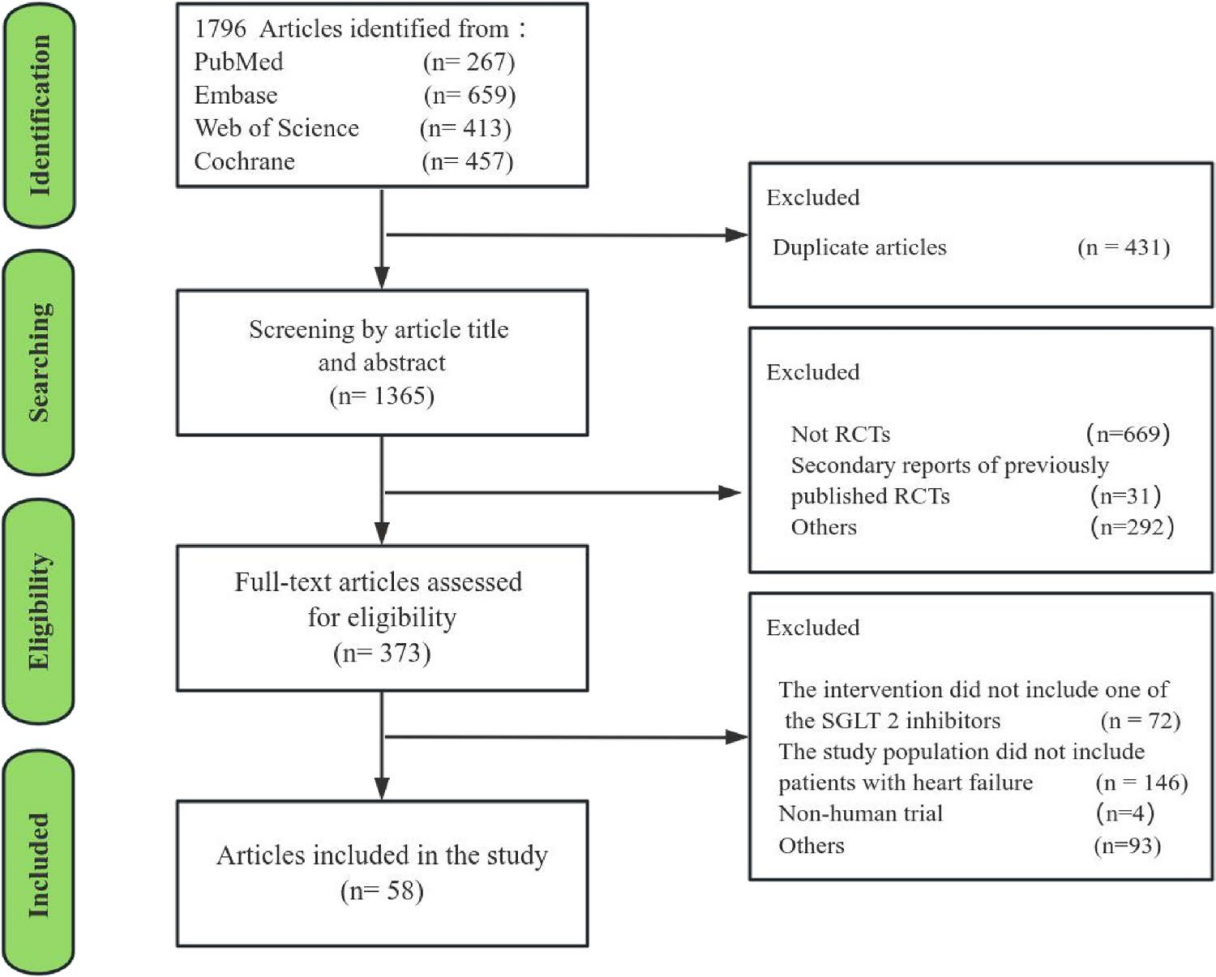
Flow chart of the selection of the 58 trials.

Out of the 1796 search results retrieved from PubMed (267), Embase (659), Web of Science (413), and Cochrane (457), 1738 were excluded based on the specified inclusion and exclusion criteria. This process entailed title/abstract and full-text screening, as depicted in Fig. 1. Ultimately, 58 RCTs that fulfilled the criteria were included in this study, as detailed in the Supplementary Material. The characteristics of the included randomized controlled trials are comprehensively detailed in Table 1. Among the 58 trials, 51 (87.93%) were published post-2020. Geographically, 31 (53.45%) of the trials were conducted in Europe, 7 (12.07%) in North America, 13 (22.41%) in Asia, and 7 (12.07%) across multiple regions. Grant funding was secured for 49 (84.48%) of the trials, 36 trials (62.07%) were commercially supported, and 31 (53.45%) were disseminated in journals boasting an impact factor of 10 or higher. The interventions documented in the trials encompassed 8 varieties of SGLT 2 inhibitors. Within these, Empagliflozin served as the trial group intervention in 28 (48.28%) trials, Dapagliflozin in 20 (34.48%), and other SGLT 2 inhibitors in 10 (17.24%) trials. The sample sizes in 39 (67.24%) of the trials were fewer than 400, and 33 (56.90%) of the trials were multicentric. Furthermore, a statistically significant difference was observed in OQS between 'Journal impact factor' (*p* = 0.01) and 'Multi-center trial' (*p* < 0.01).

**Table 1:** Trial characteristics.

Characteristic	No. of studies	CONSORT sore (mean ± SD)	F/t	*P* value
*Year of publication*				
2017–2019	7 (12.07%)	13.21 ± 3.17	− 1.49	0.14
2020–2023	51 (87.93%)	15.45 ± 3.77		
*Region in which trials were conducted*				
Europe	31 (53.45%)	14.89 ± 3.89	1.61	0.20
North America	7 (12.07%)	16.29 ± 1.82		
Asia	13 (22.41%)	14.04 ± 2.77		
Multi-Regional	7 (12.07%)	17.50 ± 5.35		
*Funding*				
Yes	49 (84.48%)	15.47 ± 3.77	1.38	0.17
No	9 (15.52%)	13.61 ± 3.47		
*Business support*				
Yes	36 (62.07%)	15.39 ± 4.03	0.54	0.59
No	22 (37.93%)	14.84 ± 3.30		
*Journal impact factor*				
< 10	27 (46.55%)	16.42 ± 3.54	2.73	0.01
≥ 10	31 (53.45%)	14.06 ± 2.99		
*Interventions*				
Empagliflozin	28 (48.28%)	15.30 ± 3.58	0.54	0.59
Dapagliflozin	20 (34.48%)	14.58 ± 3.64		
Other	10 (17.24%)	16.05 ± 4.60		
*Sample size*				
0–400	39 (67.24%)	14.49 ± 3.16	− 2.07	0.42
> 400	19 (32.76%)	16.06 ± 4.51		
*Multi-center trial*				
Yes	33 (56.90%)	16.38 ± 3.82	2.98	< 0.01
No	25 (43.10%)	13.60 ± 3.08		

### Risk of bias

**Figure 2:**
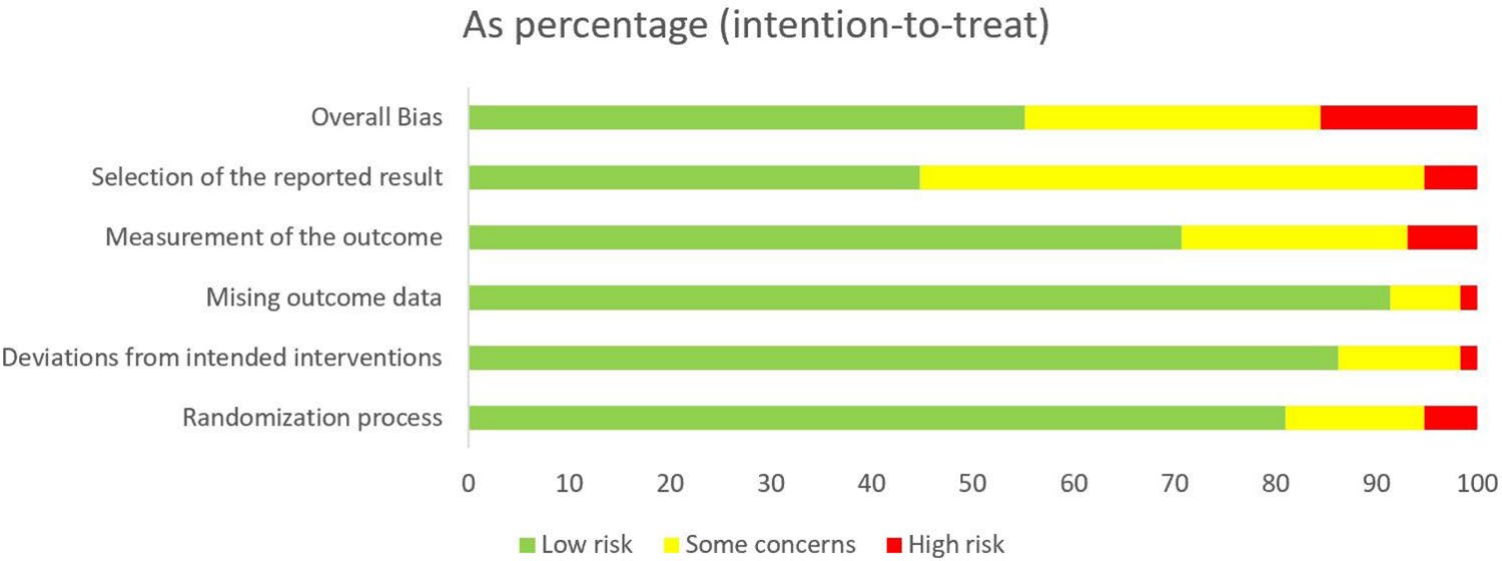
Summary of risk of bias for included trials.

All 58 included studies were subjected to analysis using the RoB 2.0 intention-to-treat checklist. In total, 55.2% of the studies were deemed to have a low risk of bias, 29.3% an unclear risk of bias, and 15.5% a high risk of bias, as illustrated in Fig. 2. Regarding the 'randomization process', 81% of the studies were assessed as having a low risk of bias, 13.8% an unclear risk of bias, and 5.2% a high risk of bias. Concerning 'deviation from intended interventions', 86.2% of the studies exhibited a low risk of bias, 12.1% an unclear risk of bias, and 1.7% a high risk of bias. Pertaining to 'missing outcome data', 91.4% of the studies were found to have a low risk of bias, 6.9% an unclear risk of bias, and 1.7% a high risk of bias. With respect to 'measurement of outcome', 70.7% of the studies were categorized as having a low risk of bias, 22.4% an unclear risk of bias, and 6.9% a high risk of bias. Lastly, in the case of 'Selection of the reported result', 44.8% of the studies demonstrated a low risk of bias, 50% an unclear risk of bias, and 5.2% a high risk of bias.

### Assessment of report quality

**Figure 3:**
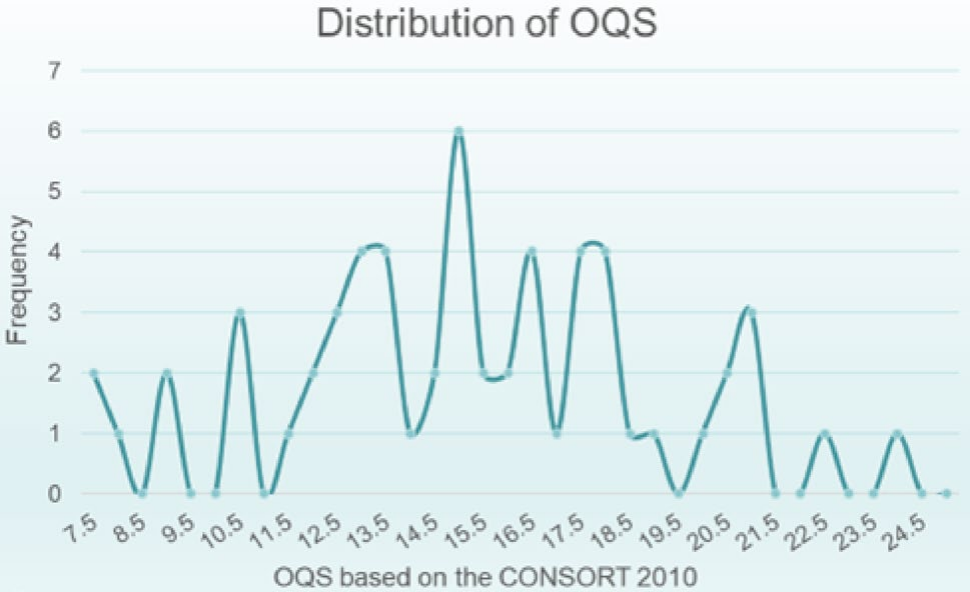
Frequency distribution of OQS for included trials.

**Figure 4:**
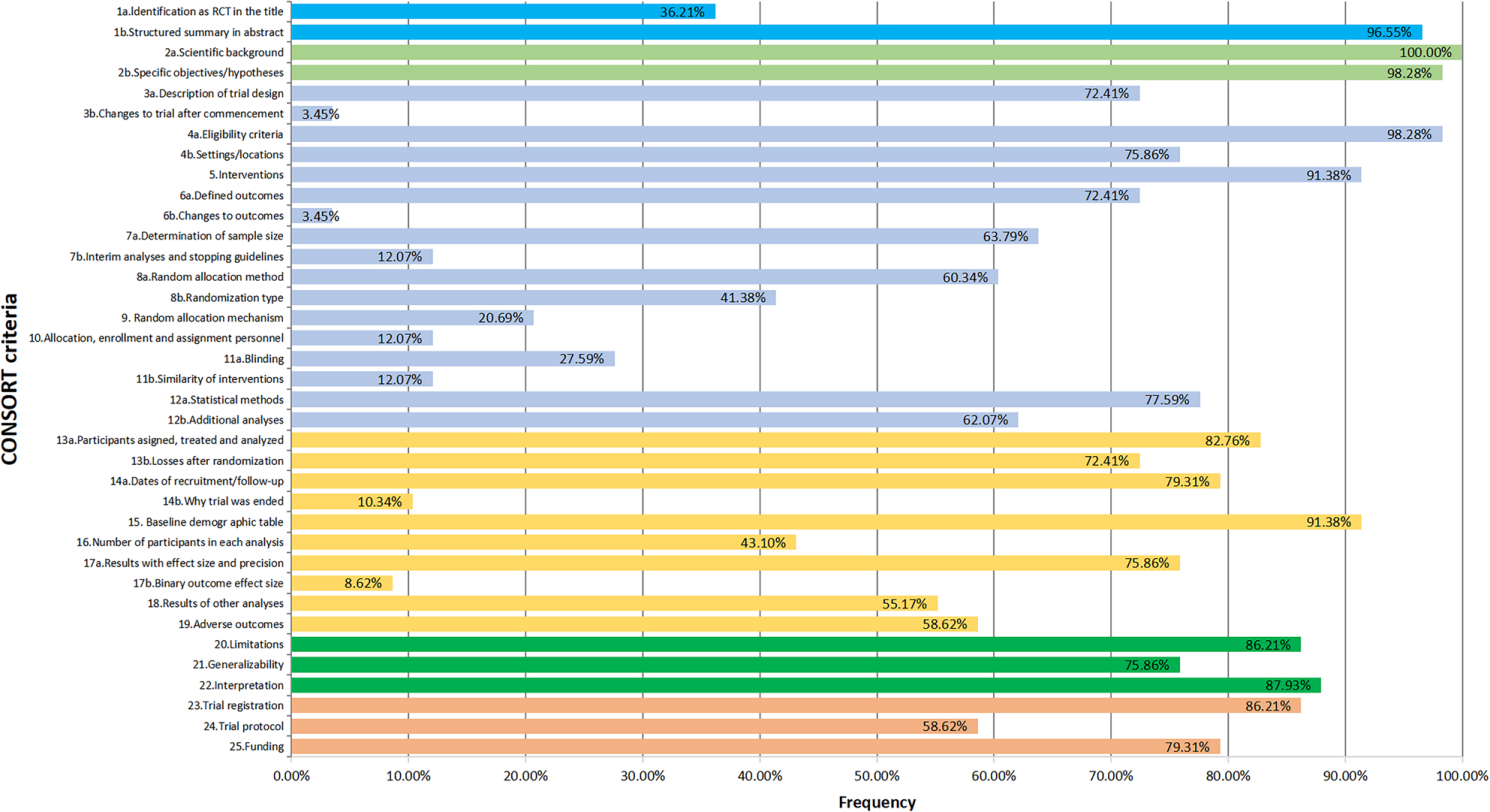
Adherence of included trials to CONSORT 2010 statement entries.

Figure 3 depicts the frequency distribution of OQS across all included studies. The median OQS corresponding to the CONSORT 2010 statement was 15, with a range from 7.5 to 24. Among all the trials, 51 (88%) exhibited an OQS ranging between 11 and 21. Figure 4 illustrates the detailed reporting of each item within the CONSORT 2010 statement. Overall, the cumulative average reporting rate for all items stood at 55.45%. A total of 9 items were reported adequately, each exceeding the 85% threshold. Within these, 4 items featured a reporting rate surpassing 95%: item 1b (56/58; 96.55%), item 2a (58/58; 100%), item 2b (57/58; 98.28%), and item 4a (57/58; 98.28%). Conversely, 7 items were reported inadequately, each falling below the 15% threshold. Within these, 3 items demonstrated a reporting rate below 10%: item 3b (2/58; 3.45%), item 6b (2/58; 3.45%), and item 17b (5/58; 8.62%).

### Factors associated with reporting quality

Table 2 delineates the results of the linear regression analysis. In the univariate model, variables such as Journal impact factor (*P* < 0.01), Sample size (*P* = 0.04), and Multi-centre trial (*P* < 0.01) demonstrated significant associations with report quality. Conversely, the multivariate analysis revealed that Year of publication (*P* = 0.04), Journal impact factor (*P* < 0.01), and Sample size (*P* = 0.01) were significantly linked to report quality. On the other hand, variables including Region of trial conduct, Funding, Control group interventions, and Multi-centre trials showed no significant associations in the multivariate analysis.

**Table 2:** Factors associated with key elements of the CONSORT guidelines.

Characteristics	Mean OQS	Univariate analysis			Multivariable analysis		
		Estimate 95% CI	t	*p*	Estimate 95% CI	t	*p*
Constant					(− 2569.99 to − 33.64)	− 2.07	0.04
Year of publication	15.18	0.16 (− 0.29 to 1.16)	1.22	0.23	0.24 (0.02 to 1.28)	2.09	0.04
*Region in which trials were conducted*							
Europe	14.89	Referent			Referent		
North America	16.29	0.12 (− 1.70 to 4.50)	0.90	0.37	0.06 (− 2.03 to 3.31)	0.49	0.63
Asia	14.04	− 0.10 (− 3.30 to1.60)	− 0.70	0.49	− 0.06 (− 2.98 to 1.83)	− 0.48	0.64
Multi-regional	17.50	0.23 (− 0.49 to 5.71)	1.69	0.10	− 0.25 (− 6.192 to 0.49)	− 1.72	0.09
*Funding*							
No	13.61	Referent			Referent		
Yes	15.47	0.18 (− 0.85 to 4.57)	1.38	0.17	0.08 (− 1.73 to 3.41)	0.66	0.51
Journal impact factor	15.18	0.58 (0.02 to 0.05)	5.27	< 0.01	0.93 (0.03 to 0.08)	5.05	< 0.01
*Interventions*							
Empagliflozin	15.30	Referent			Referent		
Dapagliflozin	14.58	− 0.09 (− 2.95 to 1.49)	− 0.66	0.51	0.12 (− 1.14 to 3.01)	0.91	0.37
Other	16.05	0.08 (− 2.05 to 3.54)	0.54	0.60	0.15 (− 1.06 to 3.95)	1.16	0.25
*Sample size*							
< 400	14.49	Referent			Referent		
≥ 400	16.06	0.27 (0.07 to 4.16)	2.07	0.04	0.46 (0.90 to 6.34)	2.68	0.01
*Multi-center trial*							
No	13.60	Referent			Referent		
Yes	16.38	0.37 (0.91 to 4.65)	2.98	< 0.01	0.18 (− 0.83 to 3.48)	1.24	0.22

## Discussion

The purpose of the CONSORT statement is to augment the transparency and caliber of reporting in research trials^9,23^. Inaccurate or insufficient reporting may impede the evaluation of results and potentially mislead policymakers. High-quality reporting facilitates peer assessment of trial quality and design, fosters study reproducibility, diminishes ambiguity, and bolsters transparency^24^. In this study, 58 RCTs underwent analysis, revealing a median CONSORT 2010 OQS of 15 (range 0–25). The overall average reporting rate across all items stood at 55.45%. These findings align with the reporting quality noted in RCTs across various medical specialties^25–27^. Clearly, there exists a significant scope for enhancing the reporting quality of RCTs on SGLT 2 inhibitors for HF, especially in the 'Methods' and 'Results' sections of the CONSORT 2010 statement. This limitation impedes the practical application of the study's findings in clinical settings.

The 2010 CONSORT Statement Adherence Survey demonstrated significant variations in project reporting rates. Certain items were comprehensively reported, including items 1b (56/58; 96.55%), 2a (58/58; 100%), 2b (59/58; 98.28%), and 4a (59/58; 98.28%). These items, pivotal in delineating the study design, necessitate accurate and detailed reporting to safeguard the credibility and replicability of the study outcomes. These elements not only constitute an integral component of the RCT report but also represent the most immediate and fundamental aspects, thereby meriting considerable emphasis in the documentation^28^. Moreover, the meticulous reporting of these items is imperative for the successful peer-review and subsequent publication of such studies.

The reporting frequency for specific items within the 2010 CONSORT statement 'Methods' and 'Results' sections is notably deficient. For instance, a mere 2 studies (3.45%) disclosed methodological alterations and their justifications subsequent to trial commencement (item 3b). Furthermore, the documentation of alterations in outcomes post-trial initiation and their rationales (item 6b) was accomplished in only 2 studies (3.45%). This paucity of reporting may stem from extensive pre-planning of most trials, the absence of substantial alterations during the trial, or the investigators' perception of these changes as insufficiently impactful to warrant reporting. Additionally, the lack of comprehensive documentation of methodological or outcome modifications, particularly in protracted or complex trials, could contribute to scant reporting. Factors such as minor adjustments to eligibility criteria during the trial to ensure an adequate sample size, or unforeseen events affecting the timing of outcome assessment, can easily be overlooked. Authors might be hesitant to reveal such alterations due to concerns that it could cast doubt on the study's integrity or outcomes in the eyes of journal editors and readers^29^.

The reporting frequency for specific items within the 'Methods' and 'Results' sections of the 2010 CONSORT statement is notably deficient. For instance, only 2 studies (3.45%) disclosed methodological alterations and their justifications subsequent to trial commencement (item 3b). Furthermore, documentation of alterations in outcomes post-trial initiation and their rationales (item 6b) was reported in only 2 studies (3.45%). This paucity of reporting may stem from extensive pre-planning of most trials, the absence of substantial alterations during the trial, or the investigators' perception of these changes as insufficiently impactful to warrant reporting. Additionally, the lack of comprehensive documentation of methodological or outcome modifications, particularly in protracted or complex trials, could contribute to scant reporting. Factors such as minor adjustments to eligibility criteria during the trial to ensure an adequate sample size, or unforeseen events affecting the timing of outcome assessment, can easily be overlooked. Authors might be hesitant to reveal such alterations due to concerns that it could cast doubt on the study's integrity or outcomes in the eyes of journal editors and readers.

In the present investigation, a scant 5 studies (8.62%) reported the binary outcome effect size (item 17b). This might stem from researchers prioritizing the reporting of statistical significance over effect size, coupled with a possible lack of requisite statistical acumen for accurate effect size calculation and disclosure. The deficiency in reporting these items is indefensible, and analogous trends are observable across various medical specialties. In an investigation by Hajibandeh et al.^30^, the caliber of reporting across 150 RCTs in vascular and endovascular surgery was scrutinized. The findings indicated that not a single article disclosed any methodological modifications post-trial commencement. Additionally, it was determined that the reporting frequencies for items 6b and 17b fell below 20%. Intriguingly, the reporting frequencies for these particular items exhibited no notable variances over time. Another analytical study by Yin et al.^31^ concentrated on 53 RCTs pertaining to COVID-19. The outcomes suggested that merely half of the studies documented the 'Binary outcome effect size'. Moreover, it was ascertained that the reporting frequency for alterations in methodology and outcomes subsequent to trial initiation was zero. The omission of reporting changes during a trial can result in consequential implications. This can impede reviewers and readers in comprehensively grasping the study process and evaluating whether all critical results have been contemplated and reported. When significant alterations are implemented in a trial but remain unreported, the outcomes may manifest bias, which remains undetected and unrectified^32^. Additionally, unreported modifications to pre-established outcome indicators can lead to interpretative errors in the results and undermine the study's reliability. Unreported outcome alterations may engender suspicions of selective reporting, wherein only advantageous or noteworthy results are disclosed, thereby affecting the comprehensive evidence base for the study field^33^. The role of effect sizes in ascertaining the clinical significance of results is paramount, and their non-disclosure may impede clinicians in precisely evaluating the genuine benefits of an intervention. Moreover, the reporting of effect sizes is indispensable for decision-making support in clinical practice and policy formulation, and the absence of such information can culminate in inadequately informed decisions^34,35^. Consequently, it is recommended that investigators meticulously document and disclose any alterations made during trials, regardless of their perceived triviality. Furthermore, researchers should prioritize trial pre-registration by systematically registering study designs, methods, etc., in public databases before initiating a trial to establish a comparable benchmark. Correspondingly, journals and reviewers should prioritize the integration of information regarding trial modifications and statistical methodologies in submitted manuscripts, and rigorously verify them during the review process. Readers and journal editors should promote fairness and inclusivity by encouraging authors to report any methodological or outcome changes that occur during the trial process, acknowledging that these changes are inherent in trials.

This study identified a concerning trend of underreporting in aspects related to randomisation and blinded implementation, as per the 2010 CONSORT statement guidelines. Among the 58 trials, merely 63.79% (37/58) referenced the method for generating random allocation, and only 41.38% (24/58) elaborated on the specific type of randomisation employed. In a similar vein, a scant 20.69% (12/58) of the trials cited the method of implementing randomised allocation, with a mere 12.07% (7/58) offering detailed implementation insights. Pertaining to blinding, 27.59% (16/58) of the trials acknowledged the use of blinding, yet only 12.07% (7/58) delineated the specifics of the blinding intervention. These findings highlight a notable deficiency in the focus on the design and implementation of trial methodologies. There are multiple factors contributing to the incomplete reporting of random allocation and blinding in research studies. Primarily, researchers may prioritize study outcomes over the importance of random allocation and blinding, resulting in inadequate reporting of these crucial elements. Secondly, the intricacies associated with implementing random allocation and blinding can present challenges. For instance, in resource-constrained settings, researchers might resort to rudimentary, informal methods rather than specialized software. Moreover, in trials with double-blind or triple-blind designs, or studies with evident intervention effects, maintaining blinding can be challenging due to information leakage and distinctive intervention characteristics. Researchers may be reticent to provide detailed information on these aspects, fearing exposure of study weaknesses that could undermine its credibility. Additionally, limitations imposed by journal article length requirements may compel researchers to omit comprehensive details on randomization and blinding from their reports.

Randomisation and blinding constitute the cornerstone principles of RCTs, instrumental in mitigating confounding factors and biased selection^36^. Nevertheless, improper randomisation may lead to sample selection bias, obscuring the discernment of intervention effects from potential confounders and adversely affecting the reliability and generalizability of the trial outcomes^37^. Likewise, inadequate blinding can introduce subjective bias in the assessment of outcomes, compromising informed decision-making and consequently impacting the reliability and internal validity of the trial findings^38^. Consequently, we advocate for targeted education and training for researchers to emphasize the importance of random allocation and blinding, coupled with reinforcing guidance on specific protocols and methods for precise reporting. Journals and academic institutions should provide more detailed instructions on randomization and blinding, such as utilizing professional randomization software, encouraging researchers to meticulously document operational details, extending the length of RCT articles, and developing reporting templates. These measures aim to assist researchers in articulating randomization and blinding specifics clearly and consistently, ultimately enhancing trial transparency, reliability, and quality. To guarantee reliability, investigators are urged to meticulously plan their randomisation and blinding strategies pre-trial, document this information on an accredited clinical trial registration platform, and rigorously adhere to the outlined plan. Furthermore, journal editors and manuscript authors are advised to employ the CONSORT statement as a benchmark for scrutinizing study reports, with a special emphasis on the thoroughness of reporting randomisation and blinding procedures.

The analysis using multivariate regression models demonstrated substantial associations between the quality of reporting and factors such as the Year of publication (*P* = 0.04), Journal impact factor (*P* < 0.01), and Sample size (*P* = 0.01). These results are consistent with prior research^39–41^, indicating an increasing acknowledgment among scholars and reviewers of the criticality of high-quality reporting over time. The rigorous peer review standards of journals with high impact factors, along with the incorporation of large sample sizes in studies, mirror a significant investment of resources such as funding, time, and expertise, directed towards guaranteeing the quality of research^42^. These observations highlight the extensive implementation of the CONSORT checklist in medical journals and suggest that increased compliance with CONSORT by researchers and journal reviewers would augment the quality of reporting in RCTs.

This study is subject to a number of limitations. Firstly, the literature searches undertaken in this study were not comprehensive due to resource limitations. Furthermore, the inclusion of only English-language publications might constrain the generalizability of our findings. Secondly, while data extraction and quality assessment were conducted by two independent authors, the subjective scoring of certain items in the CONSORT checklist introduces a possibility of measurement error. Thirdly, the assumption that each category 1 item equally impacts the OQS of every trial, assigning a maximum rating of 1 for each item, may not be entirely accurate. However, it is critical to consider that certain items might exert a greater influence than others, potentially resulting in either an overestimation or underestimation of the overall reporting or methodological quality of each trial. Consequently, this methodology carries a potential risk of selection bias.

## Conclusion

This research conducted a formal evaluation of the reporting quality of RCTs concerning SGLT 2 inhibitors for HF, adhering to the CONSORT statement guidelines. The insights gained from this study are expected to contribute to the progressive improvement of reporting quality in such trials. The outcomes of this study suggest that the overall reporting quality of RCTs examining SGLT 2 inhibitors for HF treatment remains subpar, thereby diminishing their potential utility. Consequently, it is imperative to enhance further the reporting quality of RCTs that involve SGLT 2 inhibitors for HF. Future RCTs ought to prioritize refining specific elements delineated in the CONSORT statement, especially within the 'Methods' and 'Results' sections.

### Supplementary Information

Supplementary Information.

## Data Availability

The datasets used and/or analyzed during the current study are available from the corresponding author on reasonable request.

## References

[1] Monti S, Grosso V, Todoerti M, Caporali R (2018). Randomized controlled trials and real-world data: Differences and similarities to untangle literature data. Rheumatology (Oxford).

[2] Cortegiani A, Absalom AR (2021). Importance of proper conduct of clinical trials. Br. J. Anaesth..

[3] Li W, van Wely M, Gurrin L, Mol BW (2020). Integrity of randomized controlled trials: Challenges and solutions. Fertil. Steril..

[4] Li W, Bordewijk EM, Mol BW (2021). Assessing research misconduct in randomized controlled trials. Obstet. Gynecol..

[5] Mulder R (2018). The limitations of using randomised controlled trials as a basis for developing treatment guidelines. Evid. Based Ment. Health.

[6] Plint AC (2006). Does the CONSORT checklist improve the quality of reports of randomised controlled trials? A systematic review. Med. J. Aust..

[7] Black N (1996). CONSORT. Lancet (London, England).

[8] Altman DG (2001). The revised CONSORT statement for reporting randomized trials: Explanation and elaboration. Ann. Intern. Med..

[9] Schulz KF, Altman DG, Moher D (2010). CONSORT 2010 Statement: updated guidelines for reporting parallel group randomised trials. BMC Med..

[10] Dekkers CCJ, Gansevoort RT, Heerspink HJL (2018). New diabetes therapies and diabetic kidney disease progression: The role of SGLT-2 inhibitors. Curr. Diabetes Rep..

[11] Scheen AJ (2018). Cardiovascular effects of new oral glucose-lowering agents: DPP-4 and SGLT-2 inhibitors. Circ. Res..

[12] Biegus J (2023). Impact of empagliflozin on decongestion in acute heart failure: The EMPULSE trial. Eur. Heart J..

[13] Kosiborod M (2018). Cardiovascular events associated with SGLT-2 inhibitors versus other glucose-lowering drugs: The CVD-REAL 2 study. J. Am. Coll. Cardiol..

[14] Kosiborod M (2017). Lower risk of heart failure and death in patients initiated on sodium-glucose cotransporter-2 inhibitors versus other glucose-lowering drugs: The CVD-REAL study (comparative effectiveness of cardiovascular outcomes in new users of sodium-glucose cotransporter-2 inhibitors). Circulation.

[15] Solomon SD (2022). Baseline characteristics of patients with HF with mildly reduced and preserved ejection fraction: DELIVER trial. JACC Heart Fail..

[16] Garla VV, Butler J, Lien LF (2021). SGLT-2 inhibitors in heart failure: Guide for prescribing and future perspectives. Curr. Cardiol. Rep..

[17] Dunlay SM (2019). Type 2 diabetes mellitus and heart failure: A scientific statement from the American Heart Association and the Heart Failure Society of America: This statement does not represent an update of the 2017 ACC/AHA/HFSA heart failure guideline update. Circulation.

[18] Viera AJ, Garrett JM (2005). Understanding interobserver agreement: the kappa statistic. Fam. Med..

[19] Chen YP (2017). Reporting quality of randomized, controlled trials evaluating combined chemoradiotherapy in nasopharyngeal carcinoma. Int. J. Radiat. Oncol. Biol. Phys..

[20] Ghimire S, Kyung E, Lee H, Kim E (2014). Oncology trial abstracts showed suboptimal improvement in reporting: A comparative before-and-after evaluation using CONSORT for Abstract guidelines. J. Clin. Epidemiol..

[21] He Y (2023). Evaluating the completeness of the reporting of abstracts since the publication of the CONSORT extension for abstracts: an evaluation of randomized controlled trial in ten nursing journals. Trials.

[22] Toulmonde M (2011). Quality of randomized controlled trials reporting in the treatment of sarcomas. J. Clin. Oncol..

[23] Hariton E, Locascio JJ (2018). Randomised controlled trials—the gold standard for effectiveness research: Study design: Randomised controlled trials. BJOG Int. J. Obstet. Gynaecol..

[24] Thoma A, Coroneos CJ, Eaves FF (2021). You can't see what you can't see: Transparency in RCT reporting, and the role of the CONSORT checklist. Aesthet. Surg. J..

[25] Liu M, Chen J, Wu Q, Zhu W, Zhou X (2021). Adherence to the CONSORT statement and extension for nonpharmacological treatments in randomized controlled trials of bariatric surgery: A systematic survey. Obes. Rev..

[26] Qin D (2022). The reporting and methodological quality of split-mouth trials in oral implantology: A methodological study. Clin. Oral Implants Res..

[27] Engel D (2023). Reporting quality of randomized controlled trials in prehabilitation: A scoping review. Perioper. Med. (Lond. Engl.).

[28] Lim CY, In J (2019). Randomization in clinical studies. Korean J. Anesthesiol..

[29] Prutsky GJ (2013). Initiation and continuation of randomized trials after the publication of a trial stopped early for benefit asking the same study question: STOPIT-3 study design. Trials.

[30] Hajibandeh S (2015). Reporting and methodological quality of randomised controlled trials in vascular and endovascular surgery. Eur. J. Vasc. Endovasc. Surg..

[31] Yin Y (2021). Evaluation of reporting quality of randomized controlled trials in patients with COVID-19 using the CONSORT statement. PLoS ONE.

[32] Treweek S (2018). Strategies to improve recruitment to randomised trials. Cochrane Database Syst. Rev..

[33] Tan PT, Cro S, Van Vogt E, Szigeti M, Cornelius VR (2021). A review of the use of controlled multiple imputation in randomised controlled trials with missing outcome data. BMC Med. Res. Methodol..

[34] Liu J (2022). Use of statistical methods among acupuncture randomized controlled trials was far from satisfactory. J. Clin. Epidemiol..

[35] Dwivedi AK (2022). How to write statistical analysis section in medical research. J. Investig. Med..

[36] Lancaster GA, Dodd S, Williamson PR (2004). Design and analysis of pilot studies: Recommendations for good practice. J. Eval. Clin. Pract..

[37] Armijo-Olivo S (2022). Selection, confounding, and attrition biases in randomized controlled trials of rehabilitation interventions: What are they and how can they affect randomized controlled trials results? Basic information for junior researchers and clinicians. Am. J. Phys. Med. Rehab..

[38] Hovmand OR, Poulsen ED, Arnfred S, Storebø OJ (2023). Risk of bias in randomized clinical trials on psychedelic medicine: A systematic review. J. Psychopharmacol. (Oxf. Engl.).

[39] Latronico N (2013). Quality of reporting of randomized controlled trials published in Intensive Care Medicine from 2001 to 2010. Intensive Care Med..

[40] Chen J (2018). Quality improvement in randomized controlled trial abstracts in prosthodontics since the publication of CONSORT guideline for abstracts: a systematic review. J. Dent..

[41] Liampas I, Chlinos A, Siokas V, Brotis A, Dardiotis E (2019). Assessment of the reporting quality of RCTs for novel oral anticoagulants in venous thromboembolic disease based on the CONSORT statement. J. Thromb. Thrombolysis.

[42] Walters SJ (2019). Sample size estimation for randomised controlled trials with repeated assessment of patient-reported outcomes: What correlation between baseline and follow-up outcomes should we assume?. Trials.

