# Supplementary material for: Quality reporting of randomized controlled trials on SGLT2 inhibitors for heart failure: a comprehensive assessment
Source: Sci Rep. 2024 Mar 21;14:6819. doi: 10.1038/s41598-024-57514-z (PMC10958037; doi:10.1038/s41598-024-57514-z)
Supplement: Supplementary file 1 — Supplementary Information. [file 41598_2024_57514_MOESM1_ESM.docx]

**Detailed characteristics of the included studies**

|  | Author | Journal | Year | Sample Size | Intervention in  the Experimental Group | Intervention in the Control Group | Use of Blinding | Multicenter Study |
| --- | --- | --- | --- | --- | --- | --- | --- | --- |
| 1 | Akasaka, H. et al ​​[^1^](#_ENREF_1) | Geriatrics & Gerontology International | 2022 | 68 | Ipragliflozin | Conventional treatment​​ | Single-blind (sonographers were blinded) | Yes |
| 2 | Anker, S. D. et al [^2^](#_ENREF_2) | New England Journal of Medicine | 2021 | 5988 | Empagliflozin | Placebo | Double-blind | Yes |
| 3 | Bhatt, D. L. et al [^3^](#_ENREF_3) | New England Journal of Medicine | 2021 | 10584 | Sotagliflozin | Placebo | Double-blind | Yes |
| 4 | Bhatt, D. L. et al [^4^](#_ENREF_4) | New England Journal of Medicine | 2021 | 1222 | Sotagliflozin | Placebo | Double-blind | Yes |
| 5 | Palau, P. et al [^5^](#_ENREF_5) | European Journal of Heart Failure | 2022 | 90 | Dapagliflozin | Placebo | Double-blind | No |
| 6 | Palau, P. et al [^6^](#_ENREF_6) | BMC Cardiovascular Disorders | 2017 | 64 | Dapagliflozin | Placebo | Double-blind | No |
| 7 | Cannon, C. P. et al [^7^](#_ENREF_7) | New England Journal of Medicine | 2020 | 8246 | ertugliflozin | Placebo | Double-blind | Yes |
| 8 | Carbone, S. et al [^8^](#_ENREF_8) | Diabetes Metabolic Research and Reviews | 2020 | 36 | canagliflozin | sitagliptin | Double-blind | No |
| 9 | Charaya, K. et al [^9^](#_ENREF_9) | Cardiorenal Medicine | 2023 | 285 | Dapagliflozin | Standard therapy | Open-label | No |
| 10 | Cunningham, J. W. et al [^10^](#_ENREF_10) | Journal of the American College of Cardiology | 2022 | 6263 | Dapagliflozin | Placebo | Open-label | Yes |
| 11 | Damman, K. et al [^11^](#_ENREF_11) | European Journal of Heart Failure | 2020 | 80 | empagliflozin | Placebo | Double-blind | Yes |
| 12 | de Boer, R. A. et al [^12^](#_ENREF_12) | British Journal Of Clinical Pharmacology | 2020 | 125 | licogliflozin | empagliflozinor placebo | Double-blind​ | Yes |
| 13 | Ejiri, K. et al [^13^](#_ENREF_13) | Journal of the American Heart Association | 2020 | 169 | Luseogliflozin | Voglibose | Open-labe | Yes |
| 14 | Ghanim, H. et al [^14^](#_ENREF_14) | Diabetes, Obesity and Metabolism | 2021 | 47 | Dapagliflozin | Placebo​ | Double-blind​ | No |
| 15 | Herrington, W. G. et al [^15^](#_ENREF_15) | New England Journal of Medicine | 2022 | 6609 | Empagliflozin | Placebo | Double-blind​ | Yes |
| 16 | Ilyas, F. et al [^16^](#_ENREF_16) | ESC Heart Failure | 2021​ | 19 | Dapagliflozin​ | Placebo | Double-blind​ | No |
| 17 | Emara, A. N. et al [^17^](#_ENREF_17) | European Journal of Pharmacology | 2023 | 87 | Dapagliflozin | Placebo | Double-blind​ | No |
| 18 | Januzzi, J. L., Jr. et al [^18^](#_ENREF_18) | Journal of the American College of Cardiology | 2017 | 666 | Canagliflozin | Placebo | Double-blind​ | No |
| 19 | Jensen, J. et al [^19^](#_ENREF_19) | American Heart Journal | 2022 | 120 | Empagliflozin | Placebo​ | Double-blind​ | Yes |
| 20 | Jensen, J. et al [^20^](#_ENREF_20) | American Heart Journal | 2020 | 190 | Empagliflozin | Placebo​ | Double-blind​ | Yes |
| 21 | Jensen, J. et al [^21^](#_ENREF_21) | Lancet Diabetes Endocrinol | 2021 | 119 | Empagliflozin | Placebo | Double-blind. | Yes |
| 22 | Yeoh, S. E. et al [^22^](#_ENREF_22) | European Heart Journal | 2023 | 61 | Dapagliflozin | metolazone | Open-labe | No |
| 23 | Kario, K. et al [^23^](#_ENREF_23) | Circulation Journal | 2018 | 132 | Empagliflozin | Placebo | Double-blind. | Yes |
| 24 | Katsiadas, N. et al [^24^](#_ENREF_24) | Frontiers in cardiovascular Medicine | 2021 | 110 | Dapagliflozin | antidiabetic treatment | Open-labe | No |
| 25 | Kayano, H. et al[^25^](#_ENREF_25) | Circulation Journal | 2020 | 78 | Dapagliflozin | Conventional therapy. | Open-labe | No |
| 26 | Kolwelter, J. et al[^26^](#_ENREF_26) | Clinical Research in Cardiology | 2023 | 74 | Empagliflozin | Placebo group | Double-blind. | No |
| 27 | Kosiborod, M. N. et al[^27^](#_ENREF_27) | The Lancet Diabetes & Endocrinology | 2021 | 1250 | Dapagliflozin | Placebo group | Double-blind. | No |
| 28 | Lee, M. M. Y. et al[^28^](#_ENREF_28) | Circulation | 2020 | 105 | Empagliflozin | Placebo | Double-blinded | Yes, |
| 29 | McMurray, J. J. V. et al[^29^](#_ENREF_29) | The New England Journal of Medicine | 2019 | 4744 | Dapagliflozin | Placebo | Double-blinded. | Yes. |
| 30 | McMurray, J. J. V. et al[^30^](#_ENREF_30) | Heart Failure | 2021 | 4304 | Dapagliflozin | placebo | Double-blinded | Yes |
| 31 | Mordi, N. A. et al[^31^](#_ENREF_31) | Circulation | 2020 | 23 | Empagliflozin | placebo | Double-blinded | No |
| 32 | Mustapic, I. et al[^32^](#_ENREF_32) | Journal of Clinical Medicine | 2022 | 36 | Empagliflozin or Dapagliflozin | optimal medical therapy | Single-blind | No |
| 33 | Nassif, M. E. et al[^33^](#_ENREF_33) | Circulation | 2019 | 263 | Dapagliflozin | placebo | Double-blinded | Yes |
| 34 | Nassif, M. E. et al[^34^](#_ENREF_34) | Nature Medicine | 2021 | 324 | Dapagliflozin | placebo | Double-blinded | Yes |
| 35 | Omar, M. et al[^35^](#_ENREF_35) | Journal of the American College of Cardiology | 2020 | 70 | Empagliflozin | placebo | Double-blinded | No |
| 36 | Omar, M. et al[^36^](#_ENREF_36) | Cardiovascular Diabetology | 2022 | 190 | Empagliflozin | placebo | Double-blinded | No |
| 37 | Packer, M. et al[^37^](#_ENREF_37) | The New England Journal of Medicine | 2020 | 3730 | Empagliflozin | placebo | Double-blinded | Yes |
| 38 | Petrie, M. C. et al[^38^](#_ENREF_38) | JAMA | 2020 | 4744 | Dapagliflozin | placebo | Double-blinded | Yes |
| 39 | Prochaska, J. H. et al[^39^](#_ENREF_39) | Clinical Research in Cardiology | 2023 | 144 | Empagliflozin | Placebo | Double-blind | No |
| 40 | Rau, M. et al[^40^](#_ENREF_40) | Cardiovascular Diabetology | 2021 | 44 | Empagliflozin | Placebo. | Double-blind | No |
| 41 | Reis, J. et al[^41^](#_ENREF_41) | Journal of Clinical Medicine | 2022 | 40 | Dapagliflozin | Usual medication | Open-label | No |
| 42 | Schulze, P. C. et al[^42^](#_ENREF_42) | Circulation | 2022 | 60 | Empagliflozin | Placebo | Double-blind | No |
| 43 | Singh, J. S. S. et al[^43^](#_ENREF_43) | Diabetes Care​ | 2020 | 56 | Dapagliflozin | Placebo​ | Open-label | No |
| 44 | Solomon, S. D. et al[^44^](#_ENREF_44) | New England Journal of Medicine​ | 2022 | 6263 | Dapagliflozin | Placebo​ | Double-blind trial​ | Yes |
| 45 | Biegus, J. et al[^45^](#_ENREF_45) | European Heart Journal​ | 2023 | 530 | Empagliflozin | Placebo​ | Open-label | Yes |
| 46 | Boorsma, E. M. et al[^46^](#_ENREF_46) | European Journal of Heart Failure | 2021​ | 79 | Empagliflozin | Placebo | Double-blind trial | Yes |
| 47 | Butt, J. H. et al[^47^](#_ENREF_47) | Journal of the American College of Cardiology​ | 2022​ | 6261 | Dapagliflozin | Placebo​ | Double-blind trial | Yes |
| 48 | Filippatos, G. et al[^48^](#_ENREF_48) | Circulation​ | 2022​ | 5988 | Empagliflozin | Placebo. | Double-blind | Yes |
| 49 | Lee, C. H. et al[^49^](#_ENREF_49) | Diabetes & Metabolism Journal | 2022 | 60 | Dapagliflozin | Sitagliptin | Open-label | No |
| 50 | Packer, M. et al[^50^](#_ENREF_50) | Circulation | 2021 | 3730 | Empagliflozin | Placebo | Double-blind | No |
| 51 | Packer, M. et al[^51^](#_ENREF_51) | Circulation | 2021 | 5988 | Empagliflozin | Placebo | Double-blind | No |
| 52 | Charaya, K. et al[^52^](#_ENREF_52) | Randomized Controlled Trial | 2022 | 102 | Dapagliflozin | diuretics | Double-blind | No |
| 53 | Santos-Gallego, C. G. et al[^53^](#_ENREF_53) | Journal of The American College of Cardiology | 2021 | 84 | Empagliflozin | Placebo. | Double-blind | No |
| 54 | Thiele, K. et al[^54^](#_ENREF_54) | ESC Heart Failure | 2022 | 19 | Empagliflozin | Placebo | Double-blind | No |
| 55 | Voors, A. A. et al[^55^](#_ENREF_55) | Nature Medicine | 2022 | 530 | Empagliflozin | Placebo | Double-blind | Yes |
| 56 | Ferreira, J. P. et al[^56^](#_ENREF_56) | Journal of The American College of Cardiology | 2022 | 5988 | Empagliflozin | Placebo | Double-blind | Yes |
| 57 | Hao, Z. et al[^57^](#_ENREF_57) | International Heart Journal | 2022 | 100 | Empagliflozin | Empagliflozin | Open-label | No |
| 58 | Ejiri, K. et al[^58^](#_ENREF_58) | Scientific Reports | 2022 | 157 | Luseogliflozin | Voglibose | Open-label | Yes |

**Reference**

1 Akasaka, H. *et al.* Effects of ipragliflozin on left ventricular diastolic function in patients with type 2 diabetes and heart failure with preserved ejection fraction: The EXCEED randomized controlled multicenter study. *Geriatrics & gerontology international* **22**, 298-304, doi:10.1111/ggi.14363 (2022).

2 Anker, S. D. *et al.* Empagliflozin in Heart Failure with a Preserved Ejection Fraction. *The New England journal of medicine* **385**, 1451-1461, doi:10.1056/NEJMoa2107038 (2021).

3 Bhatt, D. L. *et al.* Sotagliflozin in Patients with Diabetes and Chronic Kidney Disease. *The New England journal of medicine* **384**, 129-139, doi:10.1056/NEJMoa2030186 (2021).

4 Bhatt, D. L. *et al.* Sotagliflozin in Patients with Diabetes and Recent Worsening Heart Failure. *The New England journal of medicine* **384**, 117-128, doi:10.1056/NEJMoa2030183 (2021).

5 Palau, P. *et al.* Short-term effects of dapagliflozin on maximal functional capacity in heart failure with reduced ejection fraction (DAPA-VO(2) ): a randomized clinical trial. *European journal of heart failure* **24**, 1816-1826, doi:10.1002/ejhf.2560 (2022).

6 Palau, P. et al., Lang, C., McCrimmon, R. & Struthers, A. Does dapagliflozin regress left ventricular hypertrophy in patients with type 2 diabetes? A prospective, double-blind, randomised, placebo-controlled study. *BMC cardiovascular disorders* **17**, 229, doi:10.1186/s12872-017-0663-6 (2017).

7 Cannon, C. P. *et al.* Cardiovascular Outcomes with Ertugliflozin in Type 2 Diabetes. *The New England journal of medicine* **383**, 1425-1435, doi:10.1056/NEJMoa2004967 (2020).

8 Carbone, S. *et al.* The effects of canagliflozin compared to sitagliptin on cardiorespiratory fitness in type 2 diabetes mellitus and heart failure with reduced ejection fraction: The CANA-HF study. *Diabetes/metabolism research and reviews* **36**, e3335, doi:10.1002/dmrr.3335 (2020).

9 Charaya, K. *et al.* Impact of Dapagliflozin Treatment on Serum Sodium Concentrations in Acute Heart Failure. *Cardiorenal medicine* **13**, 101-108, doi:10.1159/000529614 (2023).

10 Cunningham, J. W. *et al.* Dapagliflozin in Patients Recently Hospitalized With Heart Failure and Mildly Reduced or Preserved Ejection Fraction. *Journal of the American College of Cardiology* **80**, 1302-1310, doi:10.1016/j.jacc.2022.07.021 (2022).

11 Damman, K. *et al.* Randomized, double-blind, placebo-controlled, multicentre pilot study on the effects of empagliflozin on clinical outcomes in patients with acute decompensated heart failure (EMPA-RESPONSE-AHF). *European journal of heart failure* **22**, 713-722, doi:10.1002/ejhf.1713 (2020).

12 de Boer, R. A. *et al.* Effects of the dual sodium-glucose linked transporter inhibitor, licogliflozin vs placebo or empagliflozin in patients with type 2 diabetes and heart failure. *British journal of clinical pharmacology* **86**, 1346-1356, doi:10.1111/bcp.14248 (2020).

13 Ejiri, K. *et al.* Effect of Luseogliflozin on Heart Failure With Preserved Ejection Fraction in Patients With Diabetes Mellitus. *Journal of the American Heart Association* **9**, e015103, doi:10.1161/jaha.119.015103 (2020).

14 Ghanim, H. *et al.* Dapagliflozin reduces systolic blood pressure and modulates vasoactive factors. *Diabetes, obesity & metabolism* **23**, 1614-1623, doi:10.1111/dom.14377 (2021).

15 Herrington, W. G. *et al.* Empagliflozin in Patients with Chronic Kidney Disease. *The New England journal of medicine* **388**, 117-127, doi:10.1056/NEJMoa2204233 (2023).

16 Ilyas, F. *et al.* Acute pleiotropic effects of dapagliflozin in type 2 diabetic patients with heart failure with reduced ejection fraction: a crossover trial. *ESC heart failure* **8**, 4346-4352, doi:10.1002/ehf2.13553 (2021).

17 Emara, A. N., Wadie, M., Mansour, N. O. & Shams, M. E. E. The clinical outcomes of dapagliflozin in patients with acute heart failure: A randomized controlled trial (DAPA-RESPONSE-AHF). *European journal of pharmacology* **961**, 176179, doi:10.1016/j.ejphar.2023.176179 (2023).

18 Januzzi, J. L., Jr. *et al.* Effects of Canagliflozin on Cardiovascular Biomarkers in Older Adults With Type 2 Diabetes. *Journal of the American College of Cardiology* **70**, 704-712, doi:10.1016/j.jacc.2017.06.016 (2017).

19 Jensen, J. *et al.* The effect of empagliflozin on contractile reserve in heart failure: Prespecified sub-study of a randomized, double-blind, and placebo-controlled trial. *American heart journal* **250**, 57-65, doi:10.1016/j.ahj.2022.04.008 (2022).

20 Jensen, J. *et al.* Twelve weeks of treatment with empagliflozin in patients with heart failure and reduced ejection fraction: A double-blinded, randomized, and placebo-controlled trial. *American heart journal* **228**, 47-56, doi:10.1016/j.ahj.2020.07.011 (2020).

21 Jensen, J. *et al.* Effects of empagliflozin on estimated extracellular volume, estimated plasma volume, and measured glomerular filtration rate in patients with heart failure (Empire HF Renal): a prespecified substudy of a double-blind, randomised, placebo-controlled trial. *The lancet. Diabetes & endocrinology* **9**, 106-116, doi:10.1016/s2213-8587(20)30382-x (2021).

22 Yeoh, S. E. *et al.* Dapagliflozin vs. metolazone in heart failure resistant to loop diuretics. *European heart journal* **44**, 2966-2977, doi:10.1093/eurheartj/ehad341 (2023).

23 Kario, K. *et al.* Twenty-Four-Hour Blood Pressure-Lowering Effect of a Sodium-Glucose Cotransporter 2 Inhibitor in Patients With Diabetes and Uncontrolled Nocturnal Hypertension: Results From the Randomized, Placebo-Controlled SACRA Study. *Circulation* **139**, 2089-2097, doi:10.1161/circulationaha.118.037076 (2019).

24 Katsiadas, N. *et al.* The effect of SGLT-2i administration on red blood cell distribution width in patients with heart failure and type 2 diabetes mellitus: A randomized study. *Frontiers in cardiovascular medicine* **9**, 984092, doi:10.3389/fcvm.2022.984092 (2022).

25 Kayano, H. *et al.* Dapagliflozin Influences Ventricular Hemodynamics and Exercise-Induced Pulmonary Hypertension in Type 2 Diabetes Patients　- A Randomized Controlled Trial. *Circulation journal : official journal of the Japanese Circulation Society* **84**, 1807-1817, doi:10.1253/circj.CJ-20-0341 (2020).

26 Kolwelter, J. *et al.* The SGLT2 inhibitor empagliflozin reduces tissue sodium content in patients with chronic heart failure: results from a placebo-controlled randomised trial. *Clinical research in cardiology : official journal of the German Cardiac Society* **112**, 134-144, doi:10.1007/s00392-022-02119-7 (2023).

27 Kosiborod, M. N. *et al.* Dapagliflozin in patients with cardiometabolic risk factors hospitalised with COVID-19 (DARE-19): a randomised, double-blind, placebo-controlled, phase 3 trial. *The lancet. Diabetes & endocrinology* **9**, 586-594, doi:10.1016/s2213-8587(21)00180-7 (2021).

28 Lee, M. M. Y. *et al.* Effect of Empagliflozin on Left Ventricular Volumes in Patients With Type 2 Diabetes, or Prediabetes, and Heart Failure With Reduced Ejection Fraction (SUGAR-DM-HF). *Circulation* **143**, 516-525, doi:10.1161/circulationaha.120.052186 (2021).

29 McMurray, J. J. V. *et al.* Dapagliflozin in Patients with Heart Failure and Reduced Ejection Fraction. *The New England journal of medicine* **381**, 1995-2008, doi:10.1056/NEJMoa1911303 (2019).

30 McMurray, J. J. V. *et al.* Effects of Dapagliflozin in Patients With Kidney Disease, With and Without Heart Failure. *JACC. Heart failure* **9**, 807-820, doi:10.1016/j.jchf.2021.06.017 (2021).

31 Mordi, N. A. *et al.* Renal and Cardiovascular Effects of SGLT2 Inhibition in Combination With Loop Diuretics in Patients With Type 2 Diabetes and Chronic Heart Failure: The RECEDE-CHF Trial. *Circulation* **142**, 1713-1724, doi:10.1161/circulationaha.120.048739 (2020).

32 Mustapic, I., Bakovic, D., Susilovic Grabovac, Z. & Borovac, J. A. Impact of SGLT2 Inhibitor Therapy on Right Ventricular Function in Patients with Heart Failure and Reduced Ejection Fraction. *Journal of clinical medicine* **12**, doi:10.3390/jcm12010042 (2022).

33 Nassif, M. E. *et al.* Dapagliflozin Effects on Biomarkers, Symptoms, and Functional Status in Patients With Heart Failure With Reduced Ejection Fraction: The DEFINE-HF Trial. *Circulation* **140**, 1463-1476, doi:10.1161/circulationaha.119.042929 (2019).

34 Nassif, M. E. *et al.* The SGLT2 inhibitor dapagliflozin in heart failure with preserved ejection fraction: a multicenter randomized trial. *Nature medicine* **27**, 1954-1960, doi:10.1038/s41591-021-01536-x (2021).

35 Omar, M. *et al.* Effect of Empagliflozin on Hemodynamics in Patients With Heart Failure and Reduced Ejection Fraction. *Journal of the American College of Cardiology* **76**, 2740-2751, doi:10.1016/j.jacc.2020.10.005 (2020).

36 Omar, M. *et al.* The effect of empagliflozin on growth differentiation factor 15 in patients with heart failure: a randomized controlled trial (Empire HF Biomarker). *Cardiovascular diabetology* **21**, 34, doi:10.1186/s12933-022-01463-2 (2022).

37 Packer, M. *et al.* Cardiovascular and Renal Outcomes with Empagliflozin in Heart Failure. *The New England journal of medicine* **383**, 1413-1424, doi:10.1056/NEJMoa2022190 (2020).

38 Petrie, M. C. *et al.* Effect of Dapagliflozin on Worsening Heart Failure and Cardiovascular Death in Patients With Heart Failure With and Without Diabetes. *Jama* **323**, 1353-1368, doi:10.1001/jama.2020.1906 (2020).

39 Prochaska, J. H. *et al.* Effects of empagliflozin on left ventricular diastolic function in addition to usual care in individuals with type 2 diabetes mellitus-results from the randomized, double-blind, placebo-controlled EmDia trial. *Clinical research in cardiology : official journal of the German Cardiac Society* **112**, 911-922, doi:10.1007/s00392-023-02164-w (2023).

40 Rau, M. *et al.* Empagliflozin does not change cardiac index nor systemic vascular resistance but rapidly improves left ventricular filling pressure in patients with type 2 diabetes: a randomized controlled study. *Cardiovascular diabetology* **20**, 6, doi:10.1186/s12933-020-01175-5 (2021).

41 Reis, J. *et al.* Dapagliflozin Impact on the Exercise Capacity of Non-Diabetic Heart Failure with Reduced Ejection Fraction Patients. *Journal of clinical medicine* **11**, doi:10.3390/jcm11102935 (2022).

42 Schulze, P. C. *et al.* Effects of Early Empagliflozin Initiation on Diuresis and Kidney Function in Patients With Acute Decompensated Heart Failure (EMPAG-HF). *Circulation* **146**, 289-298, doi:10.1161/circulationaha.122.059038 (2022).

43 Singh, J. S. S. *et al.* Dapagliflozin Versus Placebo on Left Ventricular Remodeling in Patients With Diabetes and Heart Failure: The REFORM Trial. *Diabetes care* **43**, 1356-1359, doi:10.2337/dc19-2187 (2020).

44 Solomon, S. D. *et al.* Dapagliflozin in Heart Failure with Mildly Reduced or Preserved Ejection Fraction. *The New England journal of medicine* **387**, 1089-1098, doi:10.1056/NEJMoa2206286 (2022).

45 Biegus, J. *et al.* Impact of empagliflozin on decongestion in acute heart failure: the EMPULSE trial. *European heart journal* **44**, 41-50, doi:10.1093/eurheartj/ehac530 (2023).

46 Boorsma, E. M. *et al.* Effects of empagliflozin on renal sodium and glucose handling in patients with acute heart failure. *European journal of heart failure* **23**, 68-78, doi:10.1002/ejhf.2066 (2021).

47 Butt, J. H. *et al.* Atrial Fibrillation and Dapagliflozin Efficacy in Patients With Preserved or Mildly Reduced Ejection Fraction. *Journal of the American College of Cardiology* **80**, 1705-1717, doi:10.1016/j.jacc.2022.08.718 (2022).

48 Filippatos, G. *et al.* Empagliflozin for Heart Failure With Preserved Left Ventricular Ejection Fraction With and Without Diabetes. *Circulation* **146**, 676-686, doi:10.1161/circulationaha.122.059785 (2022).

49 Lee, C. H. *et al.* Comparison of Serum Ketone Levels and Cardiometabolic Efficacy of Dapagliflozin versus Sitagliptin among Insulin-Treated Chinese Patients with Type 2 Diabetes Mellitus. *Diabetes & metabolism journal* **46**, 843-854, doi:10.4093/dmj.2021.0319 (2022).

50 Packer, M. *et al.* Effect of Empagliflozin on the Clinical Stability of Patients With Heart Failure and a Reduced Ejection Fraction: The EMPEROR-Reduced Trial. *Circulation* **143**, 326-336, doi:10.1161/circulationaha.120.051783 (2021).

51 Packer, M. *et al.* Effect of Empagliflozin on Worsening Heart Failure Events in Patients With Heart Failure and Preserved Ejection Fraction: EMPEROR-Preserved Trial. *Circulation* **144**, 1284-1294, doi:10.1161/circulationaha.121.056824 (2021).

52 Charaya, K. *et al.* Impact of dapagliflozin treatment on renal function and diuretics use in acute heart failure: a pilot study. *Open heart* **9**, doi:10.1136/openhrt-2021-001936 (2022).

53 Santos-Gallego, C. G. *et al.* Randomized Trial of Empagliflozin in Nondiabetic Patients With Heart Failure and Reduced Ejection Fraction. *Journal of the American College of Cardiology* **77**, 243-255, doi:10.1016/j.jacc.2020.11.008 (2021).

54 Thiele, K. *et al.* Empagliflozin reduces markers of acute kidney injury in patients with acute decompensated heart failure. *ESC heart failure* **9**, 2233-2238, doi:10.1002/ehf2.13955 (2022).

55 Voors, A. A. *et al.* The SGLT2 inhibitor empagliflozin in patients hospitalized for acute heart failure: a multinational randomized trial. *Nature medicine* **28**, 568-574, doi:10.1038/s41591-021-01659-1 (2022).

56 Ferreira, J. P. *et al.* Mineralocorticoid Receptor Antagonists and Empagliflozin in Patients With Heart Failure and Preserved Ejection Fraction. *Journal of the American College of Cardiology* **79**, 1129-1137, doi:10.1016/j.jacc.2022.01.029 (2022).

57 Hao, Z. & Zhang, Y. Different Doses of Empagliflozin in Patients with Heart Failure with Reduced Ejection Fraction. *International heart journal* **63**, 852-856, doi:10.1536/ihj.22-151 (2022).

58 Ejiri, K. *et al.* Effects of luseogliflozin and voglibose on high-risk lipid profiles and inflammatory markers in diabetes patients with heart failure. *Scientific reports* **12**, 15449, doi:10.1038/s41598-022-19371-6 (2022).
